# Touch perceptions across skin sites: differences between sensitivity, direction discrimination and pleasantness

**DOI:** 10.3389/fnbeh.2014.00054

**Published:** 2014-02-19

**Authors:** Rochelle Ackerley, Ida Carlsson, Henric Wester, Håkan Olausson, Helena Backlund Wasling

**Affiliations:** ^1^Department of Physiology, University of GothenburgGothenburg, Sweden; ^2^Clinical Neurophysiology, Sahlgrenska University HospitalGothenburg, Sweden

**Keywords:** affective touch, body map, C-tactile, human, psychophysics, sensory afferent, somatosensory, tactile

## Abstract

Human skin is innervated with different tactile afferents, which are found at varying densities over the body. We investigate how the relationships between tactile pleasantness, sensitivity and discrimination differ across the skin. Tactile pleasantness was assessed by stroking a soft brush over the skin, using five velocities (0.3, 1, 3, 10, 30 cm s^−1^), known to differentiate hedonic touch, and pleasantness ratings were gained. The ratings velocity-profile is known to correlate with firing in unmyelinated C-tactile (CT) afferents. Tactile sensitivity thresholds were determined using monofilament force detection and the tactile discrimination level was obtained in the direction discrimination of a moving probe; both tasks readily activate myelinated touch receptors. Perceptions were measured over five skin sites: forehead, arm, palm, thigh and shin. The assessment of tactile pleasantness over the skin resulted in a preference for the middle velocities (1–10 cm s^−1^), where higher ratings were gained compared to the slowest and fastest velocities. This preference in tactile pleasantness was found across all the skin sites, apart from at the palm, where no decrease in pleasantness for the faster stroking velocities was seen. We find that tactile sensitivity and discrimination vary across the skin, where the forehead and palm show increased acuity. Tactile sensitivity and discrimination levels also correlated significantly, although the tactile acuity did not relate to the perceived pleasantness of touch. Tactile pleasantness varied in a subtle way across skin sites, where the middle velocities were always rated as the most pleasant, but the ratings at hairy skin sites were more receptive to changes in stroking velocity. We postulate that although the mechanoreceptive afferent physiology may be different over the skin, the perception of pleasant touch can be interpreted using all of the available incoming somatosensory information in combination with central processing.

## Introduction

The experience of touch gives rise to sensations concerning both sensory (e.g., tactile sensitivity and discrimination) and emotional (e.g., pleasant, painful) aspects (McGlone et al., 2007). It is well known that tactile sensitivity varies across the skin, where the finger tips and facial skin are particularly receptive to touch (Weinstein, 1968). Differences in tactile sensitivity can be related to the underlying neurophysiology of the skin. Here, both the sensory afferent type and density affect the level of sensitivity. Skin can be divided into three main categories: glabrous skin (e.g., the thicker, non-hairy skin on the palms), hairy skin (e.g., the vast majority of the skin on the body, which contains different types of hairs), and mucocutaneous skin (e.g., the lips). In the present study, we investigate differences between glabrous and hairy skin tactile perception, focusing on the glabrous skin of the palm and on four hairy skin sites (forehead, arm, thigh, shin). The glabrous skin contains four types of myelinated mechanoreceptive afferents, namely: rapidly-adapting type 1 (Meissner), rapidly-adapting type 2 (Pacinian), slowly-adapting type 1 (Merkel), slowly-adapting type 2 (Ruffini) afferents (Vallbo and Johansson, 1984; Johnson, 2001). Hairy skin does not contain Meissner afferents, but instead contains the myelinated, rapidly-adapting hair and field mechanoreceptive afferents, and the unmyelinated C-tactile (CT) afferents (Vallbo et al., 1993, 1995, 1999). Therefore, the hairy skin of the body has both fast-conducting (myelinated Aβ fiber) and slowly-conducting (unmyelinated CT fiber) tactile systems.

CT afferents have been found all over the hairy skin, including the face (Nordin, 1990), forearm (Vallbo et al., 1993, 1999; Wessberg et al., 2003; Löken et al., 2009) and leg (Edin, 2001; Löken et al., 2007). Brain imaging studies have shown that the CT signals are initially processed in the insula cortex and not in the primary somatosensory cortex (Olausson et al., 2002, 2008; Morrison et al., 2011a; McGlone et al., 2012), where the myelinated inputs are processed (Trulsson et al., 2001; Hsiao, 2008; McGlone et al., 2012; Ackerley et al., 2012a). Furthermore, somatotopic responses have been in the insula found to CT-preferred stroking stimulation from both the arms and legs (Björnsdotter et al., 2009). This distinction in the primary processing area is important, as CTs have been implicated in the emotional processing of touch and in the regulation of homeostasis, leading to the “affective touch hypothesis” (Olausson et al., 2010). Studies on unmyelinated CT afferents have shown that they preferentially encode low force, slow stroking touch (Vallbo et al., 1993, 1999; Wessberg et al., 2003; Löken et al., 2009). Furthermore, the firing rate of CTs correlates with psychophysical ratings of tactile pleasantness, where skin stroking at velocities between 1–10 cm s^−1^ is rated as more pleasant than slower or faster stroking velocities, giving an inverted-U shape stroking velocity profile for both measures (Löken et al., 2009). No such relationship exists between the firing frequencies of myelinated afferents and pleasantness ratings (Löken et al., 2009).

Various brain imaging studies have implicated other regions in assessing the emotional value of touch over the body, including the orbitofrontal cortex (OFC) and primary somatosensory cortex (Francis et al., 1999; Rolls et al., 2003; Hua et al., 2008; Gazzola et al., 2012; McGlone et al., 2012; Voos et al., 2013). These areas may combine other sensory input (e.g., visual) and cognitive processes (e.g., previous experience, predictions), with tactile information. However, it is clear that pleasantness in touch can be felt using glabrous skin, despite its lack of CT afferents (Löken et al., 2011; Klöcker et al., 2012). Löken et al. (2009) find that in a group of 10 participants, an inverted-U velocity profile was present in pleasantness ratings from the palm, but it showed a lower overall mean compared to stroking on the arm. Löken et al. (2011) conducted a further investigation and found that the inverted-U velocity profile could be found on the arm and the palm for pleasantness ratings; however, they found that preceding stroking stimuli on the arm increased the perception of pleasantness on the palm. Therefore, the preference for slow stroking seems to be related to CT firing frequency, but this preference is still found to some extent when stroking areas of skin that do not contain CTs.

In contrast to the proposed emotional coding from CTs, the myelinated touch system provides the brain with temporally-accurate information about tactile events, such as the onset and duration of touch, as well as coding more complicated information such as force, velocity, vibration and texture (Johansson and Vallbo, 1979a; Vallbo and Johansson, 1984; Bensmaia, 2008; Johansson and Flanagan, 2009; Saal et al., 2009). These myelinated afferents contribute directly to sensory aspects of touch, including tactile sensitivity (Can you feel it?) and tactile discrimination (What are the properties of the stimulus?). The density of the myelinated afferents varies across the skin, where touch receptors are most numerous in the finger tips (Johansson, 1978; Johansson and Vallbo, 1979b; Vallbo and Johansson, 1984); hairy skin has a much lower density of these than glabrous skin (Provitera et al., 2007). CTs have never been found in glabrous skin, but their density over the body is unknown. Typical tests for tactile sensitivity and discrimination include using monofilament force detection, vibration detection, two-point discrimination, point localization and direction discrimination (Bell-Krotoski et al., 1993). These are regularly used in clinical tests and provide insights into the tactile abilities of healthy and diseased populations. Monofilament force detection is a quick and easy way of measuring tactile sensitivity at minute spots on the skin, providing a reliable and repeatable measure (Bell-Krotoski et al., 1993). Two-point discrimination has traditionally been used to investigate tactile discriminatory thresholds although the test has been criticized as it can produce variable results and is not objective (Bell-Krotoski et al., 1993). Tactile direction discrimination measures a person’s ability to differentiate the direction of an object moving across the skin (Norrsell et al., 2001). In comparison, there is no clinical test for tactile pleasantness; however, this may give a functional insight into the workings of the CT and C-fiber systems. A feasible way of testing the cortical signature of CT fiber function was proposed using controlled stroking with combined electroencephalography (Ackerley et al., 2012b), where an ultra-slow, late cortical potential was seen over more frontal areas.

Presently, we aimed to study the relationship between pleasantness and stroking velocity over five different skin sites. We predict that participants will prefer stroking around 1–10 cm s^−1^, with decreases in pleasantness for slower or faster stroking velocities, based on previous work investigating this at the arm (Löken et al., 2009, 2011). From these pleasantness stroking velocity profiles, we aim to seek differences between the glabrous palm skin and the other sites (forehead, arm, thigh, shin) where CT afferents are found, in relation to their sensitivity and discrimination properties, using their known afferent neurophysiological innervation as a basis.

## Methods

A total of 34 healthy participants (17 males; average age 25 years ± 3 SD) took part in the study, which investigated differences in tactile pleasantness, sensitivity and discrimination across the body. All participants were given basic information about the experiment and written, informed consent was obtained. The investigation conformed to local ethical approval from the University of Gothenburg and was performed in accordance with the Declaration of Helsinki. Participants were paid for participation. Pillows were used to support body sites during testing and participants were seated comfortably on an adjustable chair-bed. The participants wore glasses with side-covers to ensure they did not see the tests. We utilized three different psychophysical techniques to probe the participants’ tactile sensations over five different skin sites. Tactile sensitivity was tested using monofilament force detection, then tactile discrimination was tested using tactile direction discrimination, and finally tactile pleasantness was tested using a soft brush stroke over five velocities, known to produce differences in pleasantness ratings, per skin site. Each participant received each test in this order, although the order of skin site was randomized between participants. The participants changed into shorts and a t-shirt, so the five skin sites were accessed easily. Tactile sensations were explored over the following five skin sites: forehead (on the midline, located equidistant from the hairline and eye brows), left lower arm (located on the volar side equidistant from the elbow and wrist), left palm (in the center, equidistant from the wrist and bottom of the third finger), left thigh (ventral side, 10 cm proximal from the knee), left shin (ventral side, 15 cm distal from the knee). At sites where a lot of hair was present, the skin was lightly shaved. These sites were chosen to provide an overview of different types of touch perception over the body. Specifically, we included the palm as it does not contain CT afferents, the arm as it has been subject to previous investigations (e.g., Löken et al., 2009, 2011), the two leg sites as a comparison to the upper body, and the face as a further comparison to the limb sites. From these sites, it also covered the distal-proximal gradient i.e., lower leg-upper leg, palm-arm. This covered the main areas of the body that are generally seen (at least in warm weather), thus are socially-acceptable to be exposed. It is of interest to investigate more personal body sites, such as on the torso, however in the present experiment, we wanted to keep the more cognitive social touch element to a minimum.

### Tactile pleasantness

**Figure 1:**
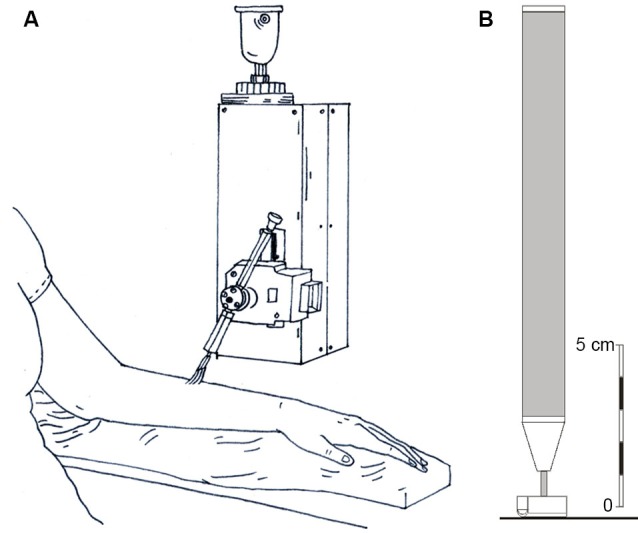
**Diagrammatic representations of the stroking stimuli and the tactile direction discrimination probe. (A)** The rotary tactile stimulator, where a soft brush was stroked at precise velocities across each skin site and psychophysical ratings of pleasantness were gained. **(B)** The probe used for the tactile direction discrimination task, which was moved across the skin over specified distances.

A rotary tactile stimulator (Figure 1A; Dancer Design, Wirral, UK) was used to deliver controlled brush strokes at a predetermined force, direction and speed to the skin sites in question, using custom-written scripts in LabVIEW (National Instruments, Austin, TX). A moving soft brush was used as a pleasant stimulus (5 cm wide goat hair artists’ brush), where previous studies have shown that velocities around 1–10 cm s^−1^ are rated as more pleasant than slower or faster velocities (Löken et al., 2009, 2011). A total of five velocities (0.3, 1, 3, 10, 30 cm s^−1^) were tested three times per skin site, in a pseudo-randomized order. The stimulator was placed over the specific skin area, with the brush end centered approximately 2 cm above the skin. The force applied by the brush was calibrated to 0.4 N. The stroking was delivered in a proximal to distal direction, apart from on the forehead where it was from right to left. After each brush stroke was delivered, the participant rated the pleasantness of the sensation using a visual analog scale with the end anchors “Unpleasant” to the left and “Pleasant” on the right. There was a 10 s pause between strokes. The output from the scale ranged from −10 (unpleasant) to +10 (pleasant). Analyses were carried out on the averages of the three stroking repeats, giving five stroking velocity pleasantness data points per skin site, per participant. Stroking velocity was transformed to log_10_ values to improve the statistical inferences and interpretation, as in previous studies (Löken et al., 2009, 2011).

### Tactile sensitivity

Participants’ sensitivity to punctate touch was tested using Von Frey monofilaments in a force detection paradigm. Five calibrated monofilaments were chosen, as determined in pre-tests, to provide a sufficient range of forces, namely: 0.7, 4, 10, 20 and 4 mN. We aimed to establish a threshold monofilament force detection level for each participant over each skin site. The method used was the increasing/decreasing detection difficulty task (Bell-Krotoski et al., 1993). Here, each monofilament was pressed five times against the selected skin site, for approximately 1 s with a 1 s gap between presses. The participant was instructed to say how many presses they felt when the experimenter asked them after the five stimuli. The monofilaments were tested first in descending force order (i.e., the task was getting more difficult), then in ascending force order. The threshold force level, for each participant per each skin site, was defined as the monofilament at which the participant could feel at least four of the five presses in both the descending and ascending order.

### Tactile direction discrimination

Tactile direction discrimination testing was carried out using a hand-held stimulator and custom-written program written in MATLAB (The Mathworks, Natick, MA). The stimulator was a small rod with a small, rounded end covered in fine, woven fabric (contact surface 0.5 cm^2^; see Figure 1B) that contacted the skin with a calibrated force of 16 g (Olausson et al., 1997; Norrsell et al., 2001). The stimulator was moved over the skin site in question in a proximal or distal direction (or right/left on the forehead) over the length of a ruler, which was inked onto the skin. The direction of travel was given in a pseudo-randomized order (Durup, 1967). The experimenter begins by moving the stimulator over 18 mm length (the middle distance) and the participant is required to state the direction of motion (“up” towards the torso or “down” away, and “left” or “right” on the forehead). If three correct answers in a row are given, the length and hence difficulty level for direction discrimination decreases to a shorter distance (10 mm, 6 mm, to a minimum of 3 mm). If an incorrect answer is given the length of travel for the next stimulation increases (32 mm, 56 mm, to a maximum of 100 mm), as per the stimulation protocol of Norrsell et al. (2001). The tactile direction discrimination threshold was calculated; after receiving 32 stimuli, the total of all the applied stimuli were added to give a value representing the response profile area (theoretical range: 18–186; see Norrsell et al., 2001 for further details). The lower the value, the more acute the tactile direction discrimination.

### Statistical analysis

All statistical testing was done using SPSS (PASW Statistics, version 18; IBM, Armonk, NY). Significances were sought at the *p* < 0.05 level, and are given up to three significant figures. Data from each task were first tested for normality of distribution using one-sample Kolmogorov-Smirnov tests. These showed that the tactile sensitivity and tactile discrimination data were not normally distributed, therefore non-parametric statistical tests were used to analyze these measures. For the tactile sensitivity and tactile discrimination data, separate Friedman’s analysis of variance (ANOVA) by ranks for related samples were used with pairwise comparisons to contrast differences between skin sites (with adjusted significance for multiple comparisons). The tactile pleasantness ratings were normally distributed (Kolmogorov-Smirnov test *p* > 0.05) and could be analyzed using parametric tests. Visual analog scales have been used for a long time to quantify subjective phenomena and even when ratings are not normally distributed, the results are often analyzed using parametric tests (for power), and show few differences to analysis using non-parametric methods (Dexter and Chestnut, 1995; Maxwell, 1978). Visual analog scales output continuous data; however, questions arise as to whether participants use the scale in similar ways. To overcome this issue, we used multi-level (mixed) modeling methods, where individual participants’ variations in ratings are taken into account. Multi-level modeling has been used for drawing comparisons in previous studies using pleasantness ratings (Essick et al., 1999, 2010). In the multi-level model, each participant’s ratings were included as random factors, using an unstructured covariance type, with the intercept included.

The effects of stroking velocity (five levels: 0.3, 1, 3, 10 and 30 cm s^−1^) and skin site (five levels: forehead, arm, palm, shin, thigh) were included as fixed factors, where main effects of each, and their interaction, were sought using maximum likelihood estimation. The gender of the participant was also included as a covariate in this top-level analysis. Where main effects for each variable were found, multi-level models were applied to seek significant differences between the levels of the stroking velocity and skin site, independently. We deal with the effects of skin site first. In the analysis of skin site effects per stroking velocity, we used multi-level modeling, with the same parameters as above for the participant effects. Further, a *post hoc* analysis was conducted to compare stroking on the palm to the other sites, using Bonferroni-corrected pairwise comparisons of the estimated marginal means. This was a planned comparison to test whether the glabrous skin of the palm (which does not contain CTs and is used more for discriminative touch) shows differences to the other hairy skin sites (which all contain CTs), as we were primarily interested in the contribution of CTs to the pleasantness ratings.

We deal with the effects of stroking velocity second; to assess the relationship between pleasantness ratings over the different velocities, per skin site, we initially conducted regression analyses. This was to probe whether the data were best fit by linear or quadratic models (i.e., whether a straight or curved line best represented increasing stroking velocity, within our range), to define a velocity-pleasantness profile, per skin site. To investigate differences in the effects of stroking velocity per skin site, we then used multi-level modeling with the same parameters as above for the participant effects. Two *post hoc* analyses were conducted using control velocities: (i) comparing the pleasantness ratings at 30 cm s^−1^ to the other velocities, and (ii) comparing the pleasantness ratings at 3 cm s^−1^ to the other velocities. This was done to assess whether the pleasantness ratings varied in comparison to (i) the stroking velocity that evokes very little CT activity (at 30 cm s^−1^ stroking there are virtually no spikes, and when they occur, it is at a low firing frequency), and (ii) the stroking velocity that is optimal for CT firing frequency (3 cm s^−1^; Löken et al., 2009). In both of these planned *post hoc* analyses, in line with related, previous work (Morrison et al., 2011a,b; McGlone et al., 2012), the stroking velocity levels in (i) and (ii) were contrasted using pairwise comparisons of the estimated marginal means, with Bonferroni corrections for multiple comparisons.

We also wanted to explore whether the results from the tactile sensitivity, discrimination and pleasantness tests were related, therefore, correlation analyses were utilized. Non-parametric correlations were used, as the tactile sensitivity and tactile discrimination data were not normally distributed. To assess these relationships, the tactile sensitivity and tactile discrimination data were entered into a correlation analysis, with the pleasantness ratings at the 3 cm s^−1^ stroking velocity. This stroking velocity was chosen to assess whether high-pleasantness stroking (cf. Löken et al., 2009) correlated with the other measures. Significant relationships were sought using two-tailed Spearman’s correlation coefficient tests.

## Results

### Tactile pleasantness

**Figure 2:**
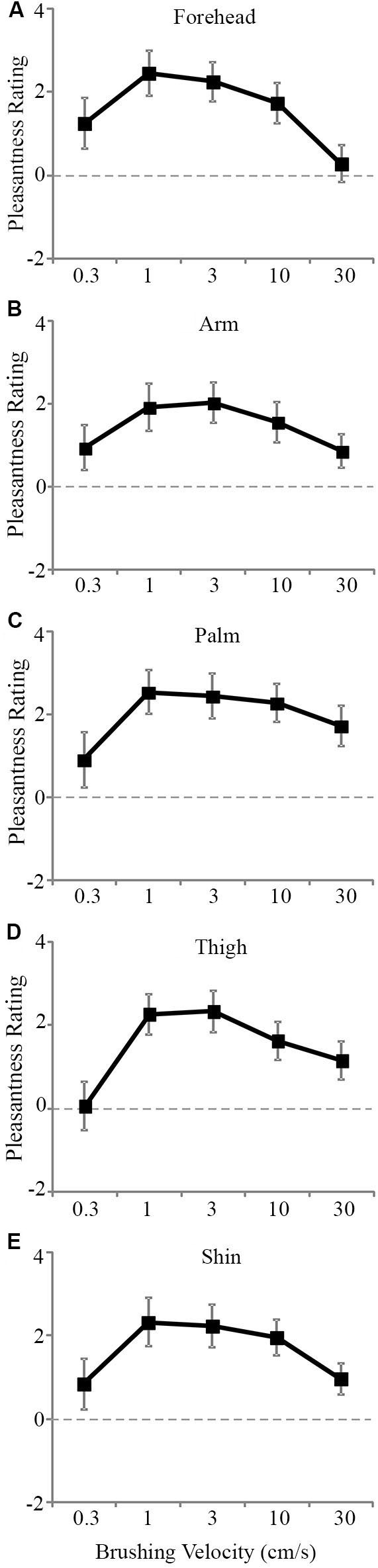
**The mean scores for pleasantness ratings at different stroking velocities over the skin sites**. A significant effect of stroking velocity overall the skin sites. The slowest (0.3 cm s^−1^) and fastest (30 cm s^−1^) were rated as less pleasant compared to the middle velocities (1–10 cm s^−1^) for the **(A)** forehead, **(B)** arm, **(C)** palm, **(D)** thigh, and **(E)** shin. The ratings scale was from −10 to +10. Error bars correspond to ±S.E.M.

The tactile pleasantness data were analyzed using multi-level modeling to uncover significant differences in the pleasantness ratings for different stroking velocities and skin sites. Main effects of both stroking velocity (*F*_4, 816_ = 24.43, *p* < 0.001) and skin site (*F*_4, 819_ = 4.61, *p* = 0.001) were found. There was no significant main effect of gender as a covariate (*p* = 0.774). The scores from the other tactile tests were also entered as covariates, but neither of these showed significant main effects with respect to the tactile pleasantness rating (tactile sensitivity, *p* = 0.374 and tactile direction discrimination, *p* = 0.240). There was no significant main interaction effect between stroking velocity and the skin site stroked (*p* = 0.686). The main effects can be seen in Figure 2A–E, where the trend was for stroking to be rated as the most pleasant at the middle velocities across all the skin sites. Although there was no significant main interaction, both variables showed significant main effects and there were multiple levels for each factor, thus further analyses were deemed appropriate to uncover the exact influences of the skin site and stroking velocity, as the main effects interaction analysis may not pick up on subtle effects over many factor levels (Field, 2009).

**Figure 3:**
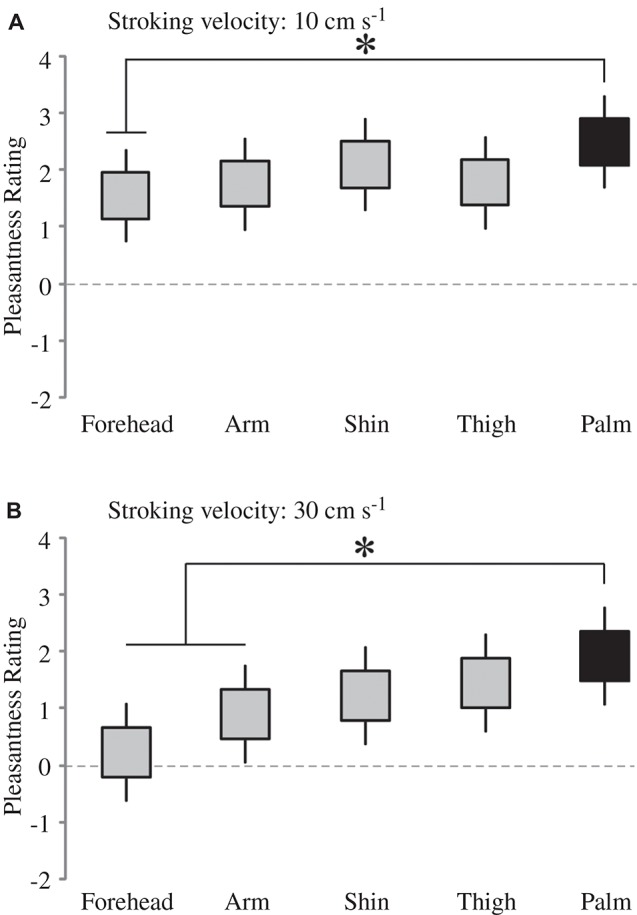
**The effect of skin site for pleasantness ratings at higher stroking velocities**. There was a significant main effect of skin site at **(A)** 10 cm s^−1^ and **(B)** 30 cm s^−1^ stroking velocities; for comparison of different skin sites at these separate velocities, the other skin sites were compared to the palm (* indicates significant differences, *p* < 0.05 to the palm ratings). The ratings scale was from −10 to +10. The upper and lower edges of the boxes relate to the ± 1 S.E.M. around the mean, and the lines are the upper and lower bound 95% confidence intervals.

Firstly, we deal with the pleasantness perception over the skin sites, at each level of stroking velocity. We conducted multi-level modeling using the ratings over the skin sites at each stroking velocity separately. We found no significant main effect of skin site at the stroking velocities of 0.3, 1 and 3 cm s^−1^, but significant main effects were found at 10 cm s^−1^ (*F*_4, 136_ = 2.48, *p* = 0.047) and 30 cm s^−1^ (*F*_4, 136_ = 5.78, *p* < 0.001). At each of these stroking velocity levels, we compared the palm as a baseline (the only site here that does not contain CT afferents) to the other skin sites. At 10 cm s^−1^ stroking velocity, pleasantness ratings from the forehead were found to be significantly lower than on the palm (*p* = 0.020; Figure 3A). At 30 cm s^−1^, pleasantness ratings from stroking the arm (*p* = 0.028) and forehead (*p* < 0.001), were significantly lower than those from the palm (Figure 3B). Overall, this indicates that the skin site differences in pleasantness ratings were present only at the faster stroking velocities.

Secondly, we deal with the pleasantness perception over the stroking velocities, at each skin site separately. Initially, we conducted regression analyses to establish the relationship between stroking velocity and perceived pleasantness, per skin site (i.e., a velocity-pleasantness profile). Here, linear and quadratic models were tested to define the stroking velocity profile. At every skin site, the profiles were all best fit by negative quadratic models, rather than linear models, giving the characteristic “inverted-U” shaped curves, as found in previous studies investigating pleasantness of different velocity stroking stimuli (Figure 2A–E, Essick et al., 1999; Löken et al., 2009, 2011). At all of the skin sites, the quadratic regressions were significant: forehead *R*^2^ = 0.07, *p* = 0.002; arm, *R*^2^ = 0.05, *p* = 0.018; palm *R*^2^ = 0.04, *p* = 0.044; shin *R*^2^ = 0.04, *p* = 0.036; thigh *R*^2^ = 0.07,* p* = 0.002 (Figure 2A–E, respectively), whereas all of the linear regressions showed no significant fit to the data. Next, using multi-level modeling, we probed the differences between pleasantness ratings over the stroking velocity levels and found a significant main effect, per skin site (all *p* < 0.001). For each skin site, we made comparisons between stroking at (i) 30 cm s^−1^ (very little CT activity) and the other velocities, and (ii) 3 cm s^−1^ (maximal CT activity) and the other velocities. Table 1 shows the results of comparison, it is evident that pleasantness ratings at the hairy skin sites showed significant increases at the middle velocities of 1 and 3 cm s^−1^, compared to the fastest stroking velocity. On the contrary, pleasantness ratings on the palm showed no significant difference over all the stroking velocities, compared to 30 cm s^−1^. Table 2 shows the results of comparison, here, we found that pleasantness ratings from stroking at 3 cm s^−1^ were not significantly different to stroking at the other middle velocities, 1 and 10 cm s^−1^, implying that these velocities give similar, increased pleasantness ratings from all over the skin.

**Table 1:** **Significant differences between pleasantness ratings from stroking at 30 cm s^−1^ compared to the other velocities, over all the skin sites**.

Velocity (compared to 30 cm s^−1^)	Pleasantness ratings profile				
	Forehead	Arm	Palm	Shin	Thigh
0.3	ns	ns	ns	ns	ns
1	*p* < 0.001	*p* = 0.015	ns	*p* = 0.018	*p* = 0.035
3	*p* < 0.001	*p* = 0.004	ns	*p* = 0.015	*p* = 0.035
10	*p* = 0.009	ns	ns	*p* = 0.046	ns

The table shows significant differences when comparing the pleasantness ratings at the fastest stroking velocity, 30 cm s^−1^, to pleasantness ratings over the other velocities, for each skin site. The data all show significant *increases* in pleasantness ratings, compared to 30 cm s^−1^, unless not significant (ns).

**Table 2:** **Significant differences between pleasantness ratings from stroking at 3 cm s^−1^ compared to the other velocities, over all the skin sites**.

Velocity (compared to 3 cm s^−1^)	Pleasantness ratings profile				
	Forehead	Arm	Palm	Shin	Thigh
0.3	*p* = 0.032	*p* = 0.001	*p* < 0.001	*p* < 0.001	*p* < 0.001
1	ns	ns	ns	ns	ns
10	ns	ns	ns	ns	ns
30	*p* < 0.001	*p* = 0.004	ns	*p* = 0.015	*p* = 0.035

The table shows significant differences when comparing the pleasantness ratings at the middle stroking velocity, 3 cm s^−1^, to pleasantness ratings over the other velocities, for each skin site. The data all show significant *decreases* in pleasantness ratings, compared to 3 cm s^−1^, unless not significant (ns).

### Tactile sensitivity

**Figure 4:**
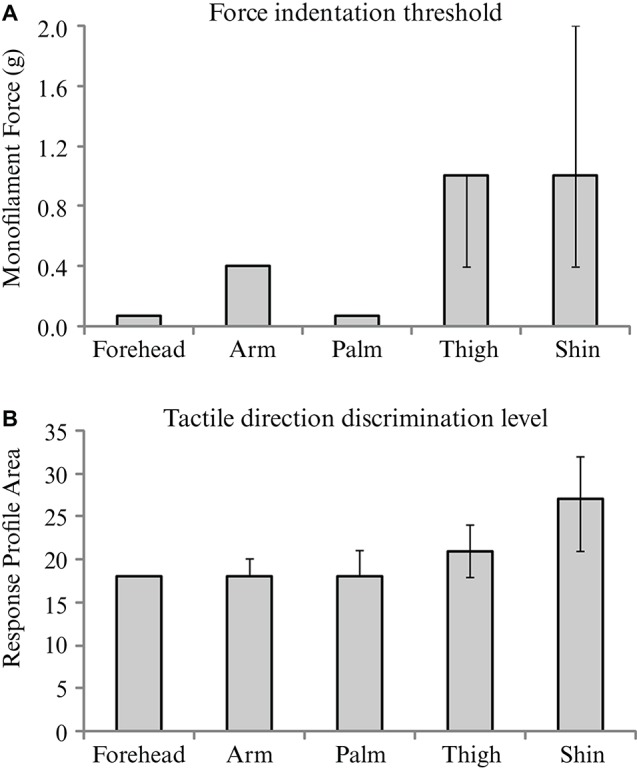
**The median values for and tactile sensitivity and discrimination over the skin**. Medians are shown for **(A)** force indentation threshold using monofilaments, and** (B)** tactile direction discrimination level for each of the five skin sites from the main experiment. Significant effects of skin site were found for both tests; see Results for individual differences between sites. Error bars indicate inter-quartile confidence intervals for the ordinal data; note that there are no inter-quartile confidence bars for the results in Figure 4A forehead, arm and palm and in Figure 4B forehead, due to there being no variation in the ranked middle 50% of ordinal scores.

Tactile sensitivity, as measured by the threshold detection level from a range of monofilament indentations, was found to differ significantly across skin sites (*p* < 0.001). The forehead and palm were the most sensitive to touch, with median detection levels of 0.07 g, corresponding to the lowest monofilament in the range tested (Figure 4A). The thigh and shin showed the least sensitivity to the tactile indentations with a median detection level of 1 g (Figure 4A). Pairwise comparisons between the threshold levels at different skin sites revealed that both the forehead and palm sites were significantly lower than at the arm, thigh and shin (all* p* < 0.01). Furthermore, the arm was found to have a significantly lower threshold for tactile sensitivity than the shin (*p* = 0.008).

### Tactile direction discrimination

The analysis of tactile direction discrimination thresholds showed significant main effects across the skin sites (*p* < 0.001). The forehead, arm and palm gave the lowest values (all medians = 18; Figure 4B), relating to acute tactile direction discrimination abilities. The skin on the shin gave the highest value (median = 27), which was significantly more than the other skin sites tested (all *p* < 0.05). The forehead was found to also have significantly better direction discrimination than the thigh (*p* = 0.036).

### Relationships between measures

Analyses were conducted to ascertain whether the results on each of the tests correlated. There was a significant, positive correlation between the score on the tactile sensitivity and the tactile discrimination tests (Spearman’s rho = 0.453, *n* = 170, *p* < 0.001). There was no significant relationship between the tactile pleasantness ratings and the other two tests (tactile sensitivity and tactile discrimination; both *p* > 0.05).

## Discussion

The present results showed that the perception of touch varies across the skin according to tactile pleasantness, sensitivity and direction discrimination. Tactile pleasantness, as measured by hedonic ratings to five velocities of stroking, was similar in profile across the skin sites investigated; however, we found that there was a subtle difference in the ratings at the glabrous palm site. Overall, the pleasantness ratings showed similar trends, where the middle velocities (1–10 cm s^−1^) were found to be more pleasant than slower (0.3 cm s^−1^) or faster (30 cm s^−1^) stroking, as seen in the stroking velocity-pleasantness profiles, where significant negative quadratic fits were found over all the skin sites. However, on closer inspection of the data, we found that the palm ratings showed a different effect to stroking velocity, although for pleasantness ratings only at the faster stroking velocities (10 and 30 cm s^−1^). We present data showing that, in essence, the pleasantness ratings on the palm at the faster stroking velocities do not decrease significantly, compared to the preferred 3 cm s^−1^. Whereas, over all the hairy skin sites tested (i.e., forehead, arm, shin and thigh), there was a significant decrease in pleasantness from stroking at 30 cm s^−1^, compared to 3 cm s^−1^ (see Tables 1, 2). Furthermore, ratings at 30 cm s^−1^ were lower for all the skin sites, compared to the palm, with the forehead and arm showing significant decreases in these ratings (Figure 3). We specifically compared the glabrous palm skin to the other sites as it does not contain CT afferents, hypothesized in signaling pleasant touch (Olausson et al., 2010), and has been previously shown to differ in the pleasantness stroking velocity profile comparing the arm and palm (Löken et al., 2009, 2011).

Human microneurographical studies have shown that the firing frequency of CT afferents, but not myelinated tactile afferents, correlates well with pleasantness ratings over a range of stroking velocities (Löken et al., 2009). The sensitivity of CT afferents to slow, gentle touch led to the CT affective touch hypothesis (Olausson et al., 2010), which postulates that the role of CTs is to signal innocuous touch between humans, aiding in social interactions and affliative behaviors. The present data lend some support the role of CTs in signaling pleasant touch, although it is clear that the pleasantness of touch can still be readily felt at skin where no CTs are present (i.e., at the glabrous palm skin in the present study, the slowest stroking velocities were less pleasant). Previous studies have found an “inverted-U” pleasantness profile to a range of stroking velocities on forearm skin, where stroking around 1–10 cm s^−1^ is preferred to slower or faster velocities (Essick et al., 1999, 2010; Löken et al., 2009, 2011; Morrison et al., 2011b). Little is known about the pleasantness stroking-velocity profile on other skin sites. Essick et al. (1999) found a similar preference for stroking at 5 cm s^−1^ on the cheek, compared to slower (0.5 cm s^−1^) and faster (50 cm s^−1^) strokes, and Essick et al. (2010) showed that pleasantness was found to be higher stroking at 5 cm s^−1^ than at 20 cm s^−1^ at the forehead, finger (glabrous skin), thigh and calf. We extend the findings from these previous studies in testing a larger range of stroking velocities at different skin sites. Löken et al. (2011) conducted an in-depth study comparing pleasantness ratings from stroking velocities between 0.1–30 cm s^−1^ on the arm and palm. They found that at both skin sites, there were decreases in pleasantness at the slowest and fastest velocities, although subtle differences were present between the pleasantness-stroking velocity profiles from the arm and palm. Similar to what we find presently, they saw a flattening of the pleasantness profile from stroking glabrous skin at the higher velocities. In our current results, the differences between CT-innervated hairy skin and non-CT-innervated glabrous palm skin were only apparent at faster stroking velocities. Stroking at 30 cm s^−1^ gives virtually no CT input (in number of spikes or firing frequency measures; Löken et al., 2009), hence this stroking velocity may lead us to infer that touch in locations with known, dense CT innervations (arm: Vallbo et al., 1993, 1999; Wessberg et al., 2003; Löken et al., 2009; face: Johansson et al., 1988; Nordin, 1990) will be less pleasant if the CTs hardly respond at all, in combination with other afference. CTs have been found in the leg (Edin, 2001; Löken et al., 2007), although we know little about their density over the whole body in humans. Animal studies suggest that the animal equivalent, C low-threshold mechanoreceptors (CLTMs) are denser in proximal locations (Liu et al., 2007; Li et al., 2011), yet this is still to be established in humans.

Previous studies testing the pleasantness perception of stroking on glabrous skin have found variable results. For example, McGlone et al. (2012) found no difference between palm and arm pleasantness ratings when stroking at 5 cm s^−1^, and Löken et al. (2011) found that recent touch on CT-innervated skin can affect pleasantness ratings for subsequent glabrous skin stroking. The processing of affective components of touch happens continuously in our everyday lives, and it is only when there are problems with the system (e.g., C-fiber denervated patients), do more obvious differences in affective processing occur (Morrison et al., 2011b), as generally, people know what a pleasant sensation should feel like and can relate to this well. Our data show that pleasant touch sensations similar to those evoked from CT-innervated skin can be experienced where CTs are not present (i.e., on the palm); hence we postulate that this sensation is based on myelinated tactile input. The glabrous skin of the hands is used for active touching, such as exploration and tactile discrimination, and thus provides the brain with a great deal of input for the evaluation of touch. This is in contrast to the hairy skin on the body, which is mainly a receiving tactile sensory organ, thus an affective touch system would be useful in the hairy skin, especially in relation to inter-personal touch. It is possible that the pleasantness sensed for soft brush stroking on the palm is based on the previous experience of gentle touch on CT-innervated areas, starting in early life. Myelinated afferents code other aspects of touch well, including force, friction and texture (Johansson and Westling, 1987; Johnson and Hsiao, 1992; Saal et al., 2009). Top-down, cognitive processes may integrate these signals to interpret how pleasant a tactile stimulus is, especially as pleasantness can be interpreted through changes in friction during exploratory touch using the glabrous skin (Klöcker et al., 2012). These mechanisms provide a way for pleasantness to be extracted centrally from myelinated afferent firing, which can be in parallel to CT input gained from hairy skin. Other affective tactile input (e.g., irritation, nociception) must also be included in the interpretation of how pleasant touch is. In the present work, the faster velocities were felt as less pleasant, and with a decrease in CT firing, other affective descriptors may encompass the sensation better. However, our scale did range from Unpleasant-Pleasant, and we found that none of the ratings in Figure 2A–E fell into the unpleasant range (i.e., less than zero). The other senses, such as audition and vision also contribute to the assessment of touch, for example, participants rate the pleasantness of visual stroking of another in a very similar way to actual stroking (Morrison et al., 2011a,b).

Brain imaging studies have shown that affective aspects of tactile stimuli on the glabrous skin of the hands elicits responses in the medial OFC (Francis et al., 1999; Rolls et al., 2003; Kida and Shinohara, 2013), which is known for its role in emotion and reward. It has been suggested that this specific activation of the OFC may be due to the evaluation of the affective component of touch, although CT activity may also activate different parts of the OFC (Hua et al., 2008; McCabe et al., 2008; McGlone et al., 2012; Voos et al., 2013). Therefore, it seems that the affective processing of touch in the OFC occurs from both glabrous and hairy skin, but it can differ under the experimental circumstances. Touch on glabrous and hairy skin does not differ much consciously (McGlone et al., 2012; Gordon et al., 2013), as slow, gentle stroking on both sites is described as pleasant. However, it is likely that the evaluation of the tactile input is very different from skin containing and not-containing CTs, where touch on hairy skin sites produces higher affective afference. This has been shown using a Touch Perception Task; here, participants used more sensory descriptors for tactile stimuli to glabrous skin, whereas they used more affective descriptors on hairy skin (Guest et al., 2011; McGlone et al., 2012; Ackerley et al., 2013), implying a role for CTs in emotional tactile evaluation.

We also compared the acuity of tactile sensitivity and direction discrimination over the same skin sites and found that these measures are correlated, but vary in a different way compared to tactile pleasantness. The skin sites demonstrating the highest tactile acuity were the forehead and palm (similar to Weinstein, 1968), which relate well to the magnified bodily representations of these areas found in the primary somatosensory cortex (Penfield and Rasmussen, 1950). The density of afferents reflects the usage of these skin surfaces: the hands are key in exploring our environment, and the face in inter-personal interactions. We attribute the tactile sensitivity and direction discrimination findings to the distribution of the myelinated Aβ tactile afferents over the skin (cf. Vallbo and Johansson, 1984; Johansson et al., 1988; Vallbo et al., 1995). The glabrous skin of the hands contains dense myelinated tactile afferents (Vallbo and Johansson, 1984) that send fast, temporally-accurate touch information to the brain, hence their high tactile acuity. The type of myelinated tactile afferent differs between glabrous and hairy skin (Vallbo et al., 1995); however, microneurographical investigations of human skin innervation have shown differences in the receptive field characteristics between hairy skin sites, for example, the skin on the face has much smaller receptive fields for rapidly-adapting afferents than on the arm (cf. Johansson et al., 1988; Vallbo et al., 1995). Therefore, the physiology shows that the innervation of the skin, even within a skin-type, is highly heterogeneous. These studies account for why the tactile sensitivity and direction discrimination was very acute on the glabrous hand and hairy forehead skin. However, these peripheral skin innervation differences do not relate very well to our present findings on tactile pleasantness.

## Conclusion

The present study, taken together with previous physiological findings on skin afferents, shows that the innervation and interpretation of incoming, innocuous tactile signals over human skin is highly heterogeneous. Tactile sensitivity and direction discrimination relate well to somatosensory cortical maps representations; however, the pleasantness of touch is a more complex percept. It is likely that tactile pleasantness is coded by CT afferents directly in the periphery, and we found subtle differences in pleasantness ratings at the higher stroking velocities between the glabrous palm skin and the other sites where CTs are present. Conversely, it is also clear that tactile pleasantness can be felt where CTs are not present (the palm), so the myelinated tactile afferent input can be readily interpreted as pleasant through central processes.

## Author contributions

All authors contributed to the conception of the work, and in drafting/revising the manuscript. All authors have approved the final version and agree to be accountable for all aspects of the work. Rochelle Ackerley designed the experiments with the help of Helena Backlund Wasling, Ida Carlsson and Henric Wester. The experiments were carried out by Ida Carlsson and Henric Wester and the data were analyzed by Rochelle Ackerley. Components of the manuscript were written by Ida Carlsson and Henric Wester, and Rochelle Ackerley drafted the full manuscript. Helena Backlund Wasling and Håkan Olausson commented on the manuscript and aided in the interpretation of the data.

## Conflict of interest statement

The authors declare that the research was conducted in the absence of any commercial or financial relationships that could be construed as a potential conflict of interest.
